# Physical Transient Photoresistive Variable Memory Based on Graphene Quantum Dots

**DOI:** 10.3390/nano12223976

**Published:** 2022-11-11

**Authors:** Lu Wang, Yukai Zhang, Peng Zhang, Dianzhong Wen

**Affiliations:** Heilongjiang Provincial Key Laboratory of Micronano Sensitive Devices and Systems, School of Electronic Engineering, Heilongjiang University, Harbin 150080, China

**Keywords:** physical transients, graphene quantum dots, flexibility, OR gates

## Abstract

Biomaterials have attracted attention as a major material for biodegradable and transient electronic devices. In this work, biocompatible gelatin-doped graphene quantum dot films are reported as active layer switching memories with good electrical properties and physical transient properties. Such nonvolatile memory devices have write-once-read-many electrical properties and a concentrated distribution of low-resistance and high-resistance states. It provides a solution for the current obstacle of resistive memory storage and computing integration. Based on the sensitivity of the device to ultraviolet light, the “OR gate” logic operation is completed. Furthermore, the active layer can be dissolved in deionized water within 15 min, and the gelatin substrate-based device can be destroyed immediately in water, indicating the potential biodegradation and physical transient properties of our fabricated device. Biocompatible memory devices are environmentally friendly, sustainable for safe storage, and low-cost, making them ideal for storage applications.

## 1. Introduction

Biomaterials have good environmental friendliness, broad application prospects, and biocompatibility, and interest in bioelectronics research is gradually increasing [1,2,3,4,5]. In addition, with the development of bioelectronic technology, the impact of electronic waste on the environment will be greatly reduced. At present, regarding bioelectronics, several biological materials have been applied, such as DNA [6,7], chitosan [8,9], protein [10,11,12], and gelatin [13,14,15]. On the other hand, transient electronics may physically disappear when needed [16,17,18,19]. Physical transient devices fabricated from biological materials can be partially or completely discarded when necessary without causing any contamination [20,21,22,23,24]. Biodegradable and transient resistive switching memories using biomaterials could enable applications in green consumer electronics and implantable biomedical devices. Among these devices, physical transient storage devices play an important role in meeting the future needs of information storage systems. Physical transient resistive switching memory, as one of the next-generation nonvolatile memories, has attracted widespread attention due to its simple structure, low cost, and low power consumption [25,26,27,28,29]. Previous studies have reported biomaterials with resistive switching and transient properties. For example, a biodegradable resistive memory with the structure W/silk fibroin/Mg was prepared with silk fibroin as the active layer, and the device disappeared after 24 h in phosphate buffer [30]. A sandwich-structured Mg/glucose/Mg resistive random access memory was fabricated using a solution method, and the device could react with water within 90 min, showing the characteristics of a physical transient [22].

GQDs have shown promising results in various electronic devices due to their edge states and variable quantum confinement effects [31,32,33], such as light-emitting diodes, photodetectors, supercapacitors, and photovoltaic solar cells. Hydrophilic nitrogen-doped GQDs (IN-GQDs) were prepared by exfoliating and decomposing graphite flakes to prepare hydrophilic nitrogen-doped GQDs. The memristor with the structure Al/IN-GQDs-albumen/ITO/Glass has an on-off current ratio of 10^4^ and can be cycled 250 times, with a hold-up time of over 10^4^ s [34]. Organic electronic synapses based on PEDOT:PSS/GQD nanocomposites were prepared by the solution method, and the current in the device decreased with increasing GQD concentration in the active layer. Applying UV light can cause changes in the properties of the quantum dots. By introducing nitrogen-doped graphene quantum dots into a graphene oxide film and irradiating the film with UV light, a memory synapse with analog resistive switching behavior was realized [35,36].

Gelatin is a biological material extracted from animal skin, bone, and other materials. Recent studies have shown that gelatin can be used as a suitable dielectric material for the fabrication of memristors with good properties. In this paper, a sandwich-structured memristor was fabricated using gelatin:GQD composites as a dielectric layer. The effect of baking temperature on the device was studied. Under the best preparation conditions, the device showed good write-once-read-many-times characteristics, a long retention time, and good stability. The response of the device to ultraviolet light was studied, and the logic operation of the “OR gate” was completed. Interestingly, the dielectric layer can be completely dissolved within 15 min, along with the disappearance of the electrical properties. Devices based on gelatin substrates can be dissolved in deionized water faster, thus achieving the effect of information confidentiality. The results show that physical transient RRAM devices can make up for the limitations of traditional RRAM devices and have broad application prospects in the field of green secure memory.

## 2. Materials and Methods

### 2.1. Preparation of the Device

RRAM devices with the structure Al/Gelatin:GQD/Al/PET were fabricated on flexible PET substrates. PET substrates were cleaned prior to device fabrication. PET was placed in acetone, absolute ethanol, and deionized water. Ultrasonic cleaning was performed for 15 min each time. Thermal evaporation was performed under a vacuum of 2 × 10^−3^ Pa to form the bottom aluminum electrode. A total of 1.5 g of gelatin was put in 60 mL of deionized water, stirred at 1000 rpm for 30 min, raising the temperature to 60 °C, and continued to be stirred for 30 min to obtain a gelatin solution. The graphene quantum dots (purchased from the manufacturer Suzhou Hengqiu Technology, concentration 1 mg/mL, purity 80%, average diameter 15 nm, and thickness 0.5–2.0 nm) and the gelatin solution were mixed in a volume ratio of 1:3. Next, the two prepared solutions were coated on an ITO-PET substrate at a low speed of 500 rpm for 5 s and a high speed of 4000 rpm for 40 s and dried at 80 °C for 10 min. Finally, thermal evaporation was performed under a vacuum of 2 × 10^−3^ Pa to form the top aluminum electrode, and the fabrication of the memristor was completed.

### 2.2. Feature Description

Two-dimensional and three-dimensional characterizations of the surface of the active layer were performed by atomic force microscopy (AFM) (Bruker, Quaschwitz, Germany). The electrical properties of the prepared RRAM were tested using a semiconductor parametric tester (Keithley 4200) (Keithley, Solon, OH, USA).

## 3. Results

**Figure 1:**
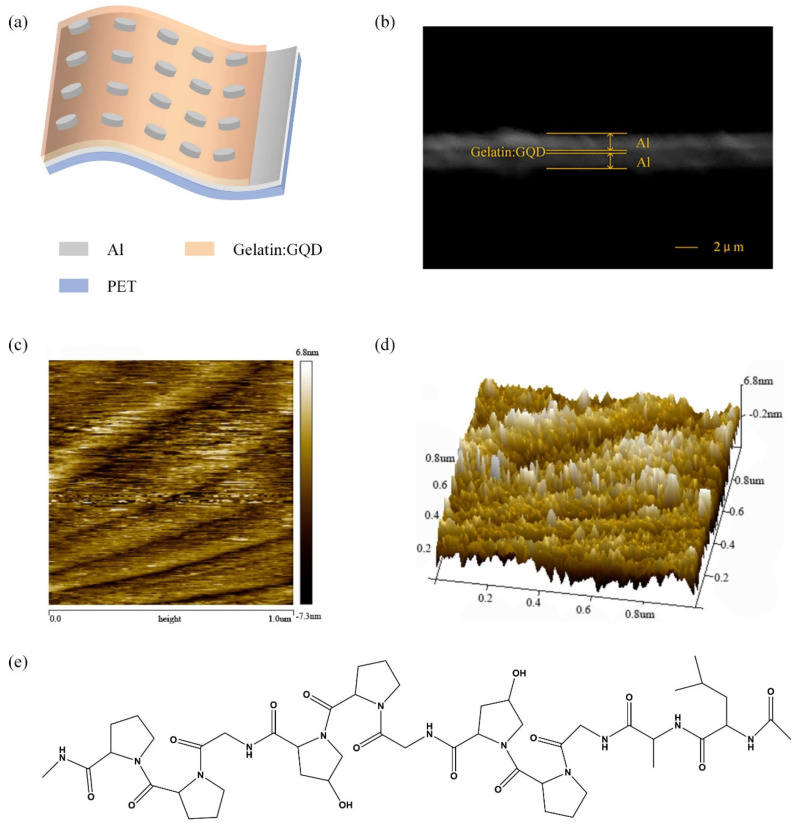
Device Al/Gelatin:GQD/Al/PET: (**a**) structure diagram and (**b**) SEM image of a cross-section. AFM images of the active layer: (**c**) two-dimensional and (**d**) three-dimensional. (**e**) Schematic diagram of the partial structure of the peptide chain in gelatin.

The structure diagram of the device is shown in Figure 1a. The prepared Al/gelatin:GQD/Al/PET device has a sandwich structure, which consists of Al electrodes, gelatin films, and Al electrodes from top to bottom. The device was characterized by scanning electron microscopy (SEM), as shown in Figure 1b. The thickness of the Al electrode is approximately 1.45 µm, and the thickness of the gelatin film is approximately 0.25 µm. As shown in Figure 1c,d, the surface flatness and smoothness of the spin-coated Gelatin:GQD layers were characterized by atomic force microscopy (AFM), with a scanning area of 1 µm × 1 µm. The results show that the surface roughness of the film is 1.88 nm, indicating that the active layer in this paper has good surface flatness. Gelatin is a material produced after the degradation of collagen, which consists of three peptide chains. Therefore, Figure 1e presents a schematic structural diagram of a partial peptide chain contained in gelatin.

**Figure 2:**
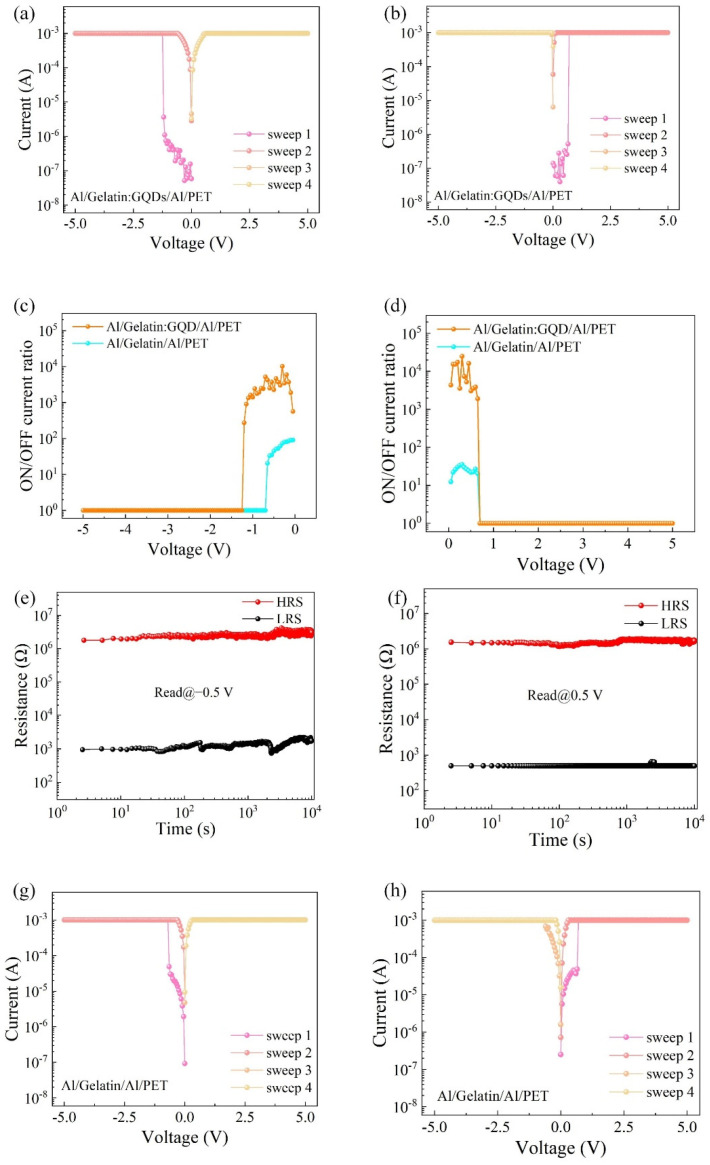
*I-V* characteristic curve of the Al/gelatin:GQD/Al/PET device: (**a**) initial negative voltage; (**b**) initial positive voltage; (**c**,**d**) switching current ratio; (**e**,**f**) holding characteristics of the *I-V* characteristic curve of the Al/gelatin/Al/PET device; (**g**) initially applied negative voltage; (**h**) initially applied positive voltage.

The *I-V* characteristics of the Al/gelatin:GQD/Al/PET device are shown in Figure 2a, where the voltage sweep direction is 0 V→−5 V→0 V→5 V→0 V. The resistance state of the initial device is the high resistance state (HRS), which can be observed in the first voltage sweep from 0 V to −1.15 V. When the voltage reaches the write voltage of the device (−1.15 V), the current increases rapidly from 1.10 × 10^−6^ A to 1.00 × 10^−3^ A, and the device switches from the HRS to the low resistance state (LRS). Then, in the subsequent three scans, the resistive state of the device always maintains LRS. Furthermore, Figure 2b shows the *I-V* characteristic curve when the scanning voltage direction is switched to 0 V→5 V→0 V→−5 V→0 V. The initial resistance state of the device is HRS. When the voltage reaches the write voltage, the device switches from HRS to LRS and maintains LRS in the subsequent scanning process. It can be seen from the above that the Al/Gelatin:GQD/Al/PET device has a write-once-read-many (WORM) characteristic. Figure 2c,d shows the ON/OFF current ratio (5.24 × 10^3^) of the Al/gelatin:GQD/Al/PET device, which is two orders of magnitude higher than the average ON/OFF current ratio (43.5) of the gelatin active layer device. In practical circuit applications, the larger the switch current ratio of the device is, the lower the false reading rate. Therefore, adding GQDs to the active layer of the device improves the ON/OFF current ratio of the device and expands the application range of the device. To test the data retention capability of the Al/Gelatin:GQD/Al/PET device, the total test time was set to 10^4^ s, and the sampling interval was 2.5 s. Figure 2e shows the retention characteristics of a read voltage of −0.50 V when a negative voltage is initially applied. Figure 2f is the holding characteristic curve of the read voltage of 0.50 V under the initial application of a positive voltage. The test results show that the device has no obvious attenuation at 10^4^ s, indicating that the device has good retention characteristics. Figure 2g,h shows the *I-V* characteristic curves of the Al/gelatin/Al/PET device initially applied with negative voltage and positive voltage, respectively. Similarly, the initial resistance state of the device is HRS. After the external voltage reaches the writing voltage, the resistance state of the device is switched from HRS to LRS, and LRS was maintained in the subsequent three scans.

**Figure 3:**
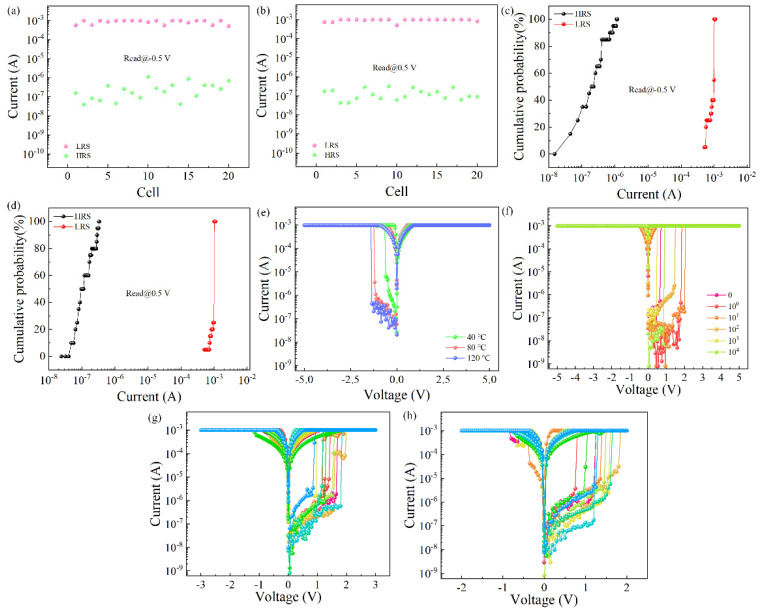
Yield rate of the Al/Gelatin:GQD/Al/PET device: (**a**) initial negative voltage applied; (**b**) initial positive voltage applied to the *I-V* characteristic curve of the Al/gelatin:GQD/Al/PET device. (**c**) Resistance accumulation probability at −0.5. (**d**) Resistance accumulation probability at −0.5. (**e**) Different drying temperatures. (**f**) Different bending times. (**g**) Scanning voltage 3 V~−3 V. (**h**) Scanning voltage 2 V~−2 V.

On this basis, the yield rate of the Al/Gelatin:GQD/Al/PET device was tested. The 20 cells of the device were tested for electrical characteristics, and the resistance values of the HRS and LRS of the device were read at 0.5 V and −0.5 V, respectively, as shown in Figure 3a,b. Figure 3c,d correspond to the current cumulative probability distribution diagram. When a negative voltage is initially applied, the coefficient of variation of the high-resistance state is 0.97, and the coefficient of variation of the low-resistance state is 0.21; when a positive voltage is initially applied, the coefficient of variation of the high-resistance state is 0.63, and the coefficient of variation for the low-resistance state is 0.14. The results show that the high and low resistance states of the device remain stable. Compared with a device with undoped graphene quantum dots, the overall switching current of the device is still significantly improved. The gap between the HRS and LRS of the 20 units of the whole device is small, indicating that the device has good consistency. The electrical properties of the memristors fabricated with active layers at different drying temperatures were studied. As shown in Figure 3e, the devices were dried at different temperatures, and the electrical properties were WORM. However, the electrical properties of the active layer at a drying temperature of 40 °C are poor, and the ON/OFF current ratio is not significantly improved. Considering that the device dried at 80 °C can achieve the expected effect, the drying temperature in the study is 80 °C. The device was fabricated on a flexible substrate, and the robustness of the device was tested. The device was bent with an exponential number of bending times. The results are shown in Figure 3f. The device can still maintain the electrical characteristics of write-once read-many after 10^4^ bending cycles. It is proven that the device can be applied in flexible usage scenarios. As shown in Figure 3g,h reducing the scanning range of the device still exhibits the electrical characteristics of WORM. The device operates normally in the scan range of −2 V to 2 V, showing that the device can work normally in the low scan voltage range and has the potential of low power consumption. The threshold voltage distributions under the write-once-many-read memory characteristic of all 20 cells on the device were counted, as shown in Figure 3f. When a negative voltage was initially applied, the average threshold voltage was −1.02 V, and the coefficient of variation was −0.47; when a positive voltage was initially applied, the average threshold voltage was 1.37 V, and the coefficient of variation was 0.22. The above results show that the device has a stable threshold voltage.

**Figure 4:**
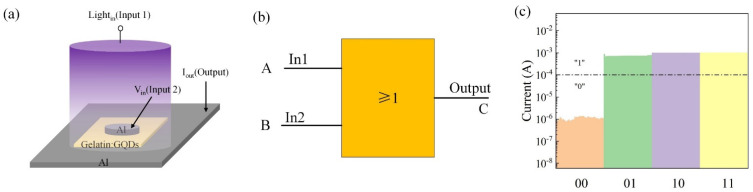
(**a**) Schematic diagram of applying UV light. (**b**) “OR” gate symbol. (**c**) Current response of the unit device under the action of photoelectric and electrical signals.

The Al/gelatin:GQD/Al/PET produced in this study is not only a voltage-controlled device but also a device that can switch its resistance state from a high-resistance state to a low-resistance state when exposed to UV light. Electron−hole pairs are generated in GQDs, and the holes migrate to the surface, which facilitates the photodesorption process of the adsorbed oxygen ions. The unpaired electrons left behind after the migration of the photogenerated holes enhance the free carrier concentration in the active layer and increase the photocurrent. The device can function as a logic or gate. Figure 4a shows a schematic diagram of the device applying UV illumination. Figure 4b shows an OR gate with a logical OR function. The two terminals of “A” and “B” are the input terminals for the electrical signal and optical signal, respectively. When the signals are input to logic devices, they are output from the “C” terminal after processing. It can be concluded that a single abovementioned device can realize the “OR” gate function, thereby reducing the complexity of integration and circuit consumption. Based on the sensitivity of GQDs to UV light, Figure 4c and Table 1 show the current response values of the Al/Gelatin:GQD/Al/PET device in the optical signal, electrical signal, and optical and electrical signal. A device with an output current greater than or equal to 10^−4^ A corresponds to a logic value of “1”, and at less than 10^−4^ A, the logic state corresponds to a “0”. The test results show that when a single electrical signal or a single optical signal is input to the device, the current of the device is approximately 1.00 × 10^−3^ A, corresponding to logic state “1”. In addition, the photoelectric time signal is applied to the device together, and the output current is approximately 1.08 × 10^−3^ A, which also corresponds to state “1”. From the table, it can be concluded that a single device can complete the operation of a logical “OR gate”.

**Figure 5:**
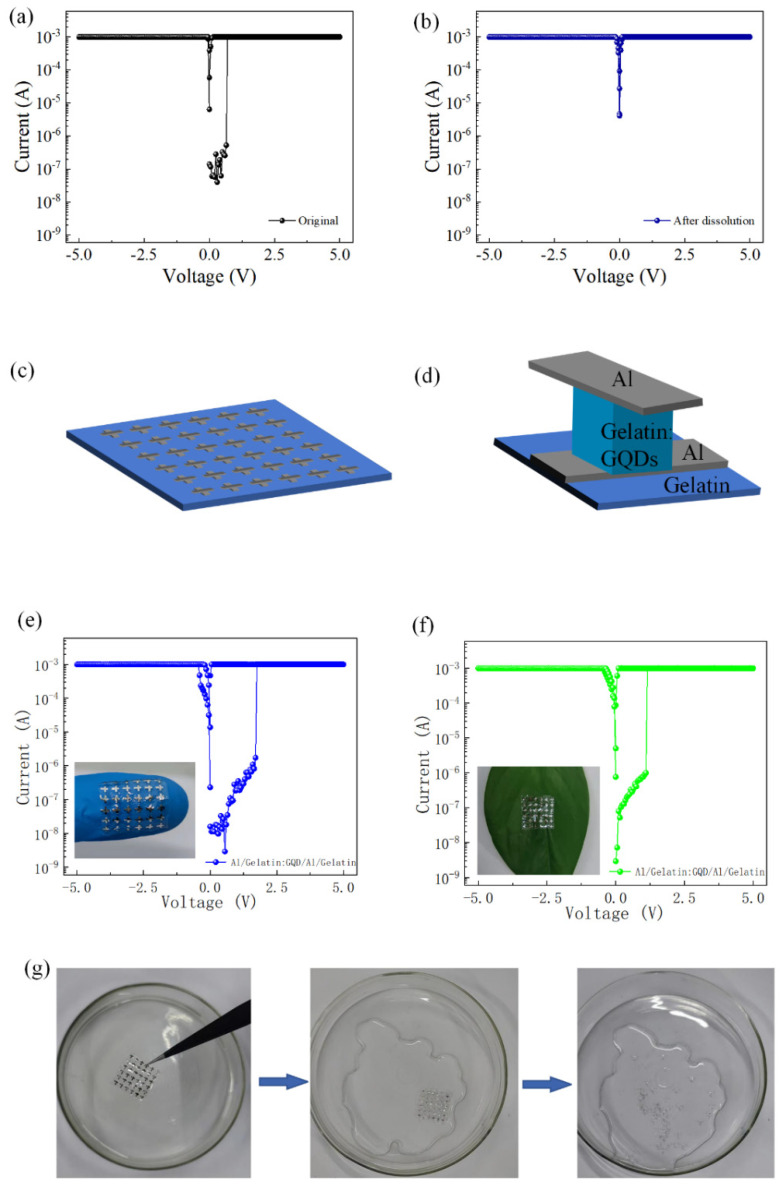
*I-V* characteristic curve of the Al/Gelatin:GQD/Al/PET device: (**a**) before immersion; (**b**) Al/Gelatin:GQD/Al/Gelatin. (**c**) Schematic diagram of the device after immersion for 15 min; (**d**) enlarged view of the structure of Al/Gelatin: *I-V* characteristic curve of the GQD/Al/Gelatin (**e**) placed on a finger (**f**) and placed on green leaf. (**g**). The active layer of the device was dissolved in deionized water.

The as-prepared gelatin:GQD films were immersed in deionized water and showed physical transient properties (Figure 5a). After soaking for several seconds, the active layer film has no obvious change. In deionized water, the film completely disappeared after approximately 15 min, as observed by light reflection. The electrical properties of the Al/gelatin:GQD/Al/PET memory device before and after dissolution were further investigated to evaluate the physical transient electronic behavior, as shown in Figure 5b. After the Al/gelatin:GQD/Al/PET device is dissolved in deionized water, its high- and low-resistance windows disappear, and the switching of the resistance state cannot be completed.

When the flexible PET was replaced with gelatin as the substrate, other preparation conditions remained unchanged, and the fabricated device structure was Al/gelatin:GQD/Al/gelatin. The structure diagram and partially enlarged pictures of the device are shown in Figure 5c,d. The device is strippable and can work on different substrates. As shown in Figure 5e,f, the stripped devices were placed on fingers and green leaves, and their electrical properties were tested. The results show that the electrical device under peeling can still complete the operation of WORM. When the device with the structure of Al/gelatin:GQD/Al/gelatin was placed in deionized water, as shown in Figure 5g, it was found that the device immediately dissolved and could no longer complete the original electrical function. These results suggest that memory devices using gelatin materials have the potential for physical transient electronic device applications and as green and secure data storage systems.

**Figure 6:**
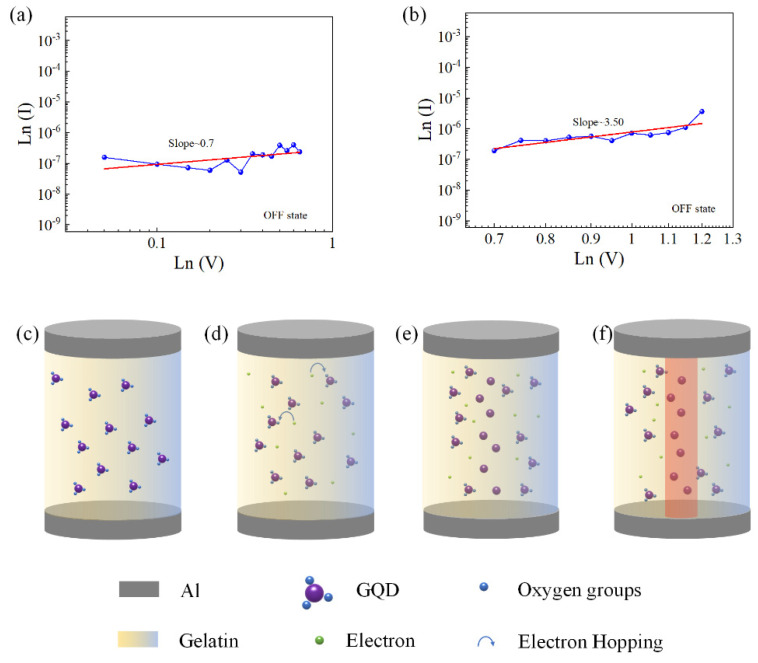
(**a**,**b**) Schematic diagram of the conduction mechanism of the device with a double logarithmic curve in the OFF state of the device. (**c**) The trapping center is in an unoccupied state. (**d**) The injected carriers are trapped by the GQD. (**e**) The trap is completely filled. (**f**) Forming conductive paths.

To further explore the current conduction mechanism of the Al/gelatin:GQD/Al/PET device, the typical *I-V* characteristic curve was redrawn in the double logarithmic coordinate, and the curve was fitted and analyzed, as shown in Figure 6a,b. The fitted slope of the LRS of the device is approximately 1, which is consistent with the ohmic conduction mechanism. In the low voltage region, the fitting slope of the HRS of the device is approximately 1. When the voltage increases, the fitting slope satisfies I∝V^n^, so the current transport mechanism of the device satisfies the SCLC conduction mechanism.

A schematic diagram of the conduction mechanism of the device is shown in Figure 6c–f. The GQDs distributed in gelatin can act as capture centers. Therefore, the introduction of GQDs in the active layer increases the density of the traps in the active layer, the high resistance state resistance of the device increases, and the ON/OFF current ratio increases. When no voltage is applied, the distribution of traps in the active layer is random (Figure 6c). When a voltage is applied to the device, in the low voltage state, thermally generated carriers cause ohmic conduction. As the voltage increases, the traps begin to be filled with carriers injected under the electric field (Figure 6d). When the applied voltage reaches the set voltage (V_set_), the traps are completely filled with carriers (Figure 6e). This leads to an increase in the injected carriers in the active layer, forming conductive paths (Figure 6f). The device switches from the OFF state to the ON state. Due to the accumulation of a large number of electrons in the active layer, an internal electric field is formed. When the device is reverse biased, due to the protection of the internal electric field, the charges in the traps are hardly neutralized or released, and the conduction path does not break. Therefore, the device exhibits a WORM memory effect.

## 4. Conclusions

In conclusion, we developed RRAM devices with physically transient properties in the Al/Gelatin:GQD/Al/PET and Al/Gelatin:GQD/Al/Gelatin structures. The device has a switching current ratio greater than 10^3^ and a retention time exceeding 10^4^ s. Based on the sensitivity of the GQDs to ultraviolet light, the logical operation of the “OR gate” is completed. Furthermore, the physical transient properties of the Gelatin:GQD-based RRAM devices were demonstrated by the active layer thin films that dissolved in deionized water within 15 min, with the gelatin substrate devices completely dissolving in deionized water. These results show a potential option for a low-cost, biodegradable ReRAM based on Gelatin:GQD for transient electronics and secure memory applications.

## Figures and Tables

**Table 1:** Data of Current response of the unit device under the action of photoelectric and electrical signals.

Input		Output	
Opt.	Ele.	Current (A)	Logic
0	0	1.06 × 10^−6^	0
0	1	7.28 × 10^−4^	1
1	0	1.00 × 10^−3^	1
1	1	1.00 × 10^−3^	1

## Data Availability

Not applicable.
